# Supplementary material for: meQTL mapping in the GENOA study reveals genetic determinants of DNA methylation in African Americans
Source: Nat Commun. 2023 May 11;14:2711. doi: 10.1038/s41467-023-37961-4 (PMC10175543; doi:10.1038/s41467-023-37961-4)
Supplement: Supplementary file 1 — Supplementary Information [file 41467_2023_37961_MOESM1_ESM.pdf]

# Supplementary Information

# meQTL mapping in the GENOA study reveals genetic determinants of DNA methylation in African Americans

Lulu Shang[1], Wei Zhao[2], Yi Zhe Wang[2], Zheng Li[1], Jerome J. Choi[3], Minjung Kho[2], Thomas H. Mosley[4], Sharon L.R. Kardia[2], Jennifer A. Smith[2,†], Xiang Zhou[1,†]

1. Department of Biostatistics, School of Public Health, University of Michigan, Ann Arbor, MI, 48109
2. Department of Epidemiology, School of Public Health, University of Michigan, Ann Arbor, MI, 48109
3. Population Health Sciences, University of Wisconsin-Madison School of Medicine and Public Health, Madison, WI 53726
4. Memory Impairment and Neurodegenerative Dementia (MIND) Center, University of Mississippi Medical Center, Jackson, MS, 39126

[†] Corresponding authors

**This file includes:**
Supplementary Figures 1-21
Supplementary Tables 1-9
Supplementary References

## Supplementary Figures

**Supplementary Figure 1. Workflow for identifying eQTLs, meQTLs, and co-localized eGene-meCpG pairs.**

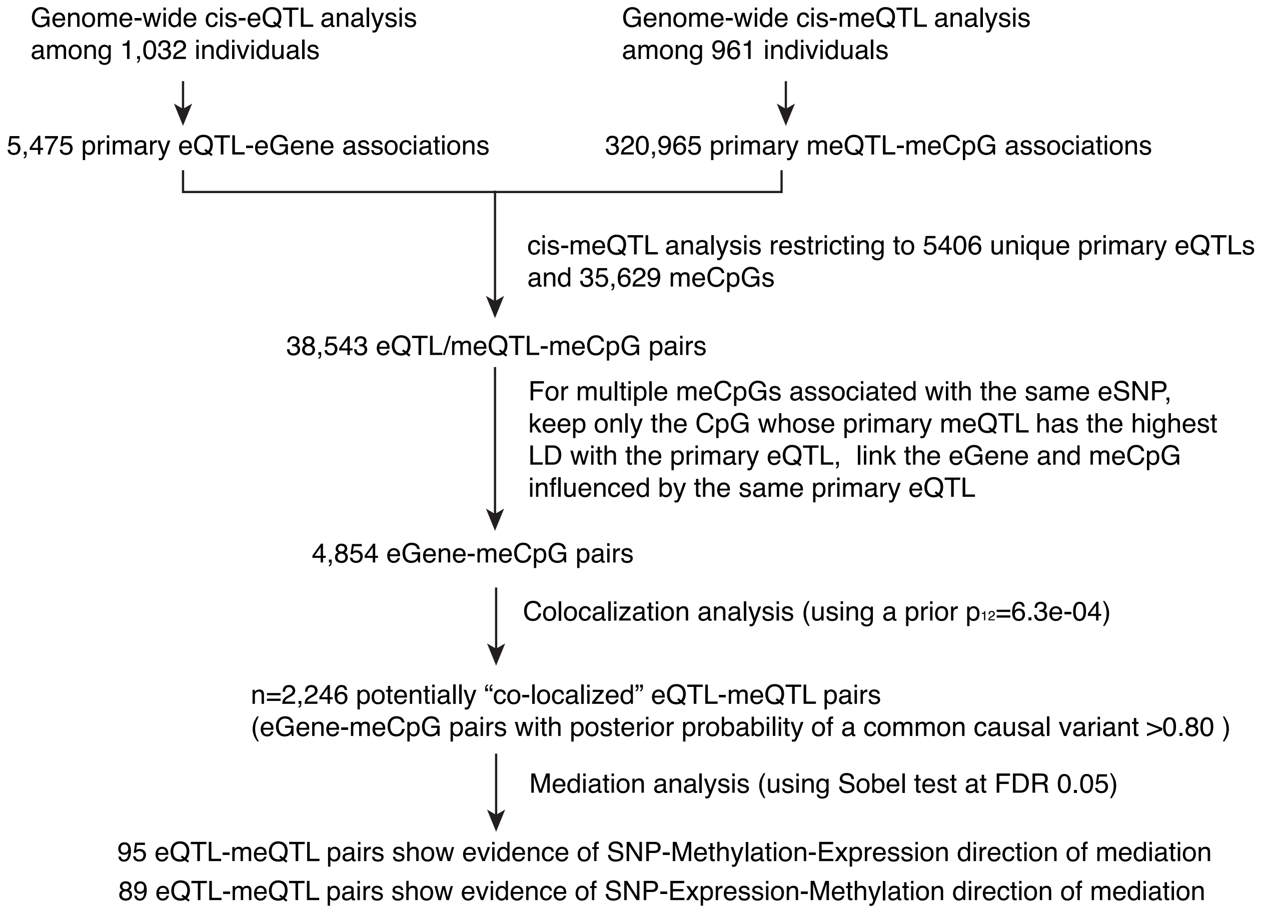

**Supplementary Figure 2**. **Odds ratio of hypo- and hyper- methylated CpG sites, together with the 95% CI computed in different annotated genomic regions. a** Hypomethylated CpG sites are enriched in genetic conserved regions such as CpG islands and TSS200 regions. **b** The hypermethylated regions are more enriched in genetically dynamic regions such as intergenic region, gene body and open seas. Bar graphs show odds ratios; error bars show 95% CIs. CIs were estimated by the Fisher's exact method. Statistics were computed based on a sample size of n = 961 and for 728,578 CpG sites.

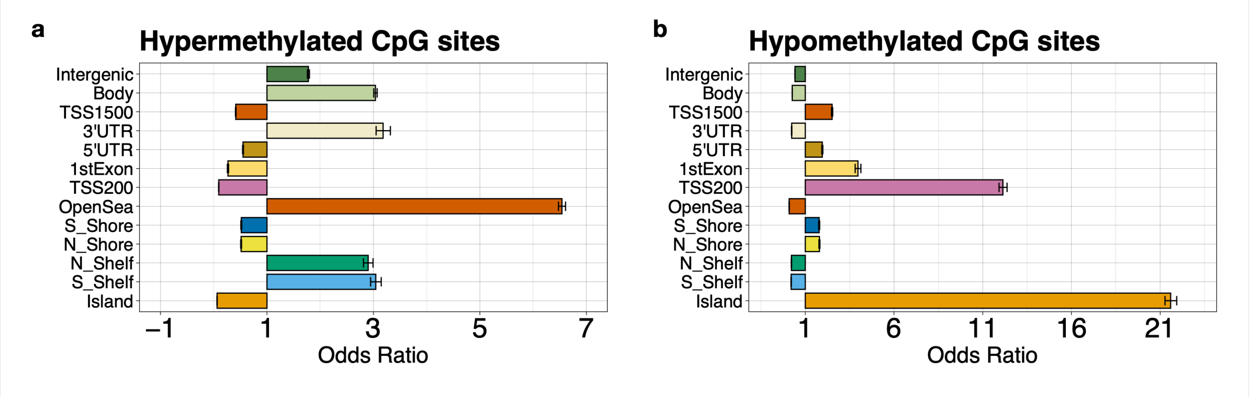

**Supplementary Figure 3. Top examples of meQTLs.** X-axis: genotype; Y-axis: normalized methylation levels. In the boxplots, the center line, box limits and whiskers denote the median, upper and lower quartiles, and 1.5× interquartile range, respectively. Statistics were computed based on a sample size of n = 961.

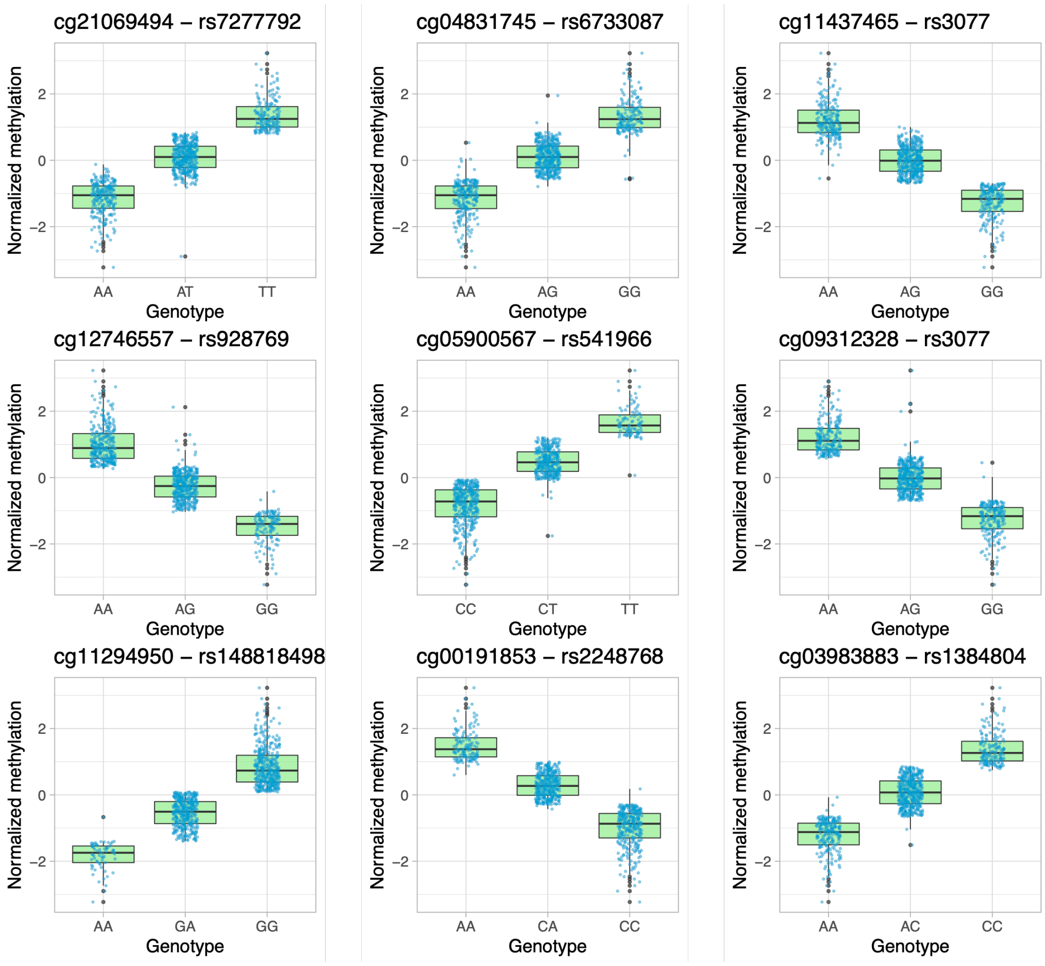

**Supplementary Figure 4. Number of meCpGs and meQTLs detected using different meQTL mapping window sizes on chromosome 22.**

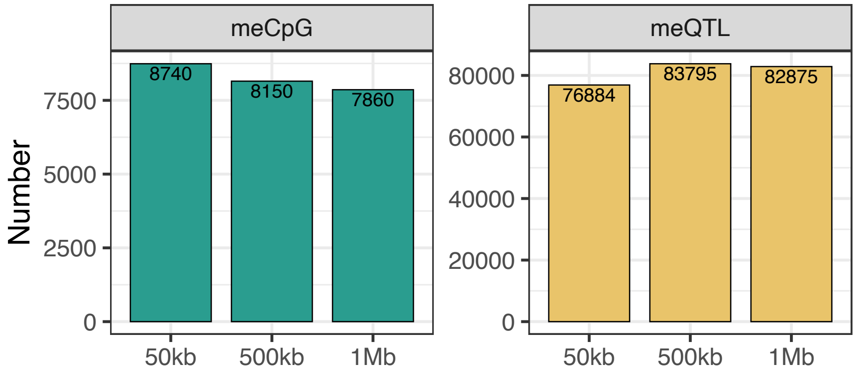

**Supplementary Figure 5. The distribution of meQTLs with respect to their associated CpG sites on chromosome 22.** The x-axis is the distance between the meQTL and its associated CpG site. meQTL mapping analysis was carried out using a window size of 1Mb.

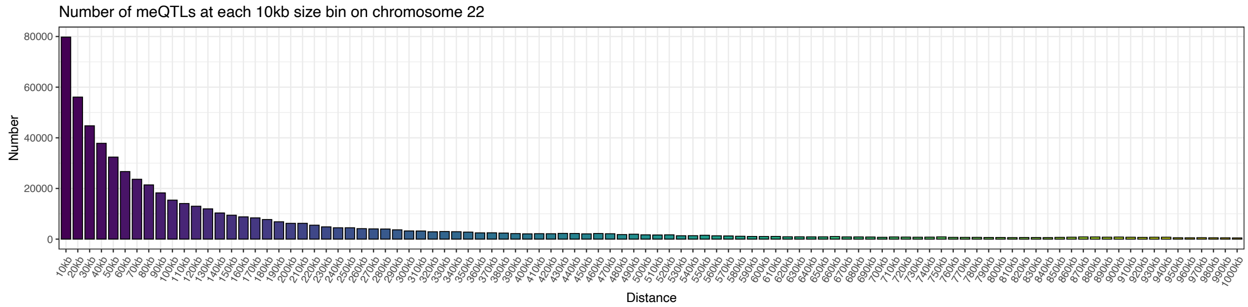

**Supplementary Figure 6. Heritability estimation with different meQTL mapping window sizes on chromosome 22. a-c** Heritability estimation with meQTL mapping window sizes at 50kb, (**a**) 500kb (**b**), and 1Mb (**c**) on chromosome 22. In the boxplots, the center line, box limits and whiskers denote the median, upper and lower quartiles, and 1.5× interquartile range, respectively. Statistics were computed based on a sample size of n = 961 and for 728,578 CpG sites.

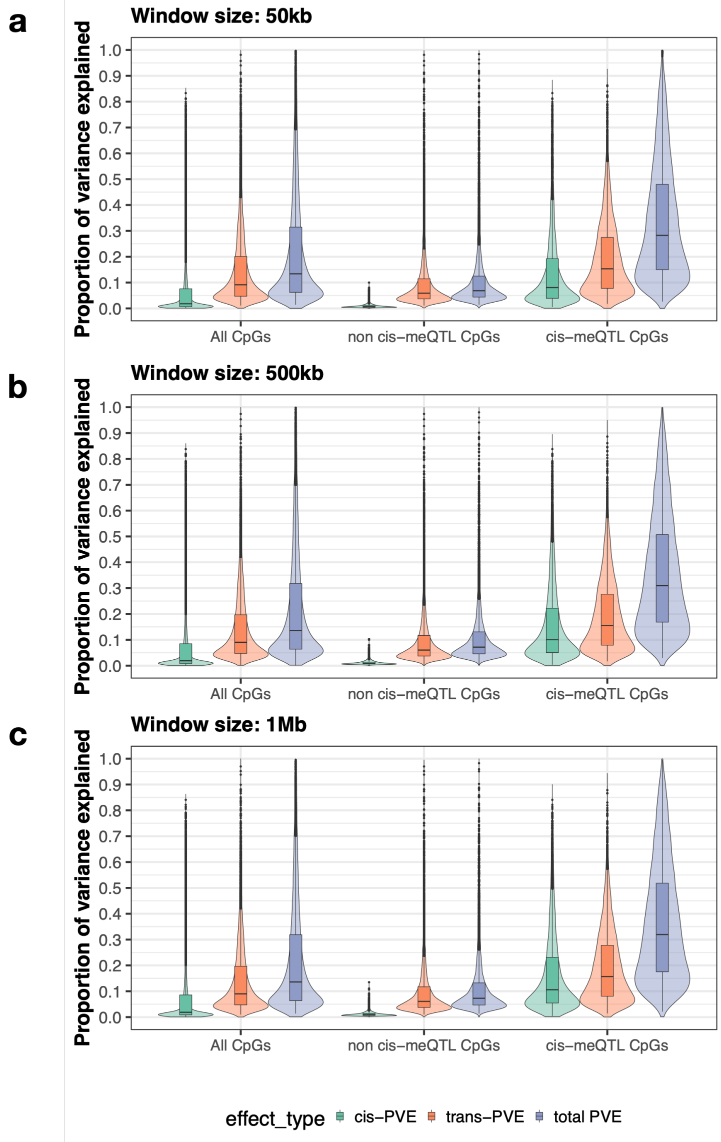

**Supplementary Figure 7. The median PVE estimates obtained with different meQTL mapping window sizes on chromosome 22.** The x-axis labels window sizes. The three column panels are median PVE (SNP heritability) for CpG sites containing cis-meQTLs, CpG sites without cis-meQTLs, and all CpG sites, respectively. The three row panels are cis-PVE, trans-PVE and total PVE, respectively. All PVE estimations are calculated for CpG sites on chromosome 22.

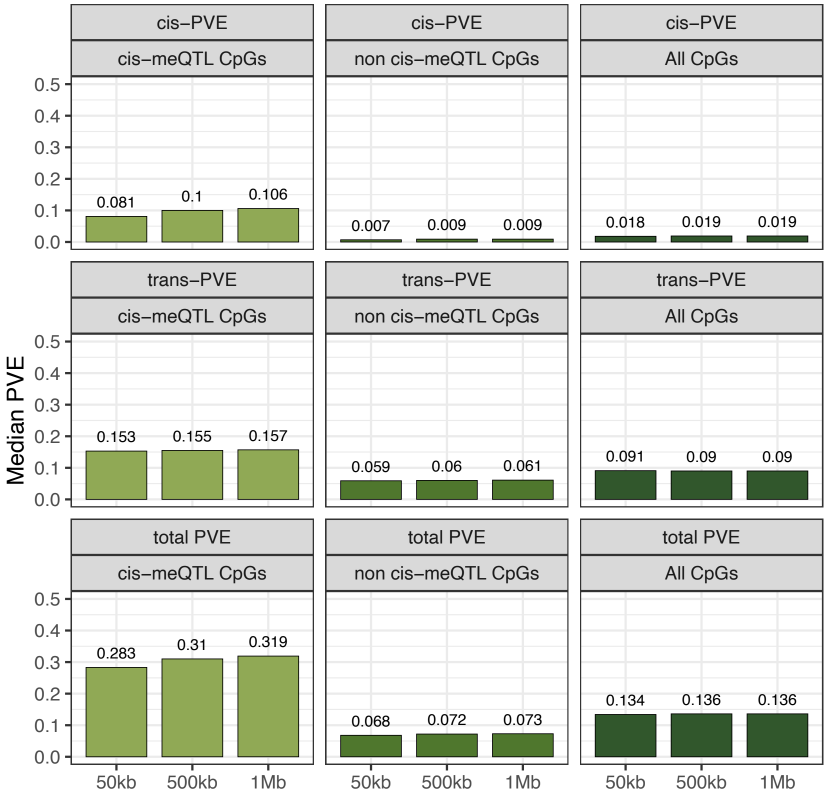

**Supplementary Figure 8. Heritability estimation with different meQTL mapping window sizes are highly consistent with each other. a-f** Comparison of PVE (SNP heritability) estimations between different window sizes.

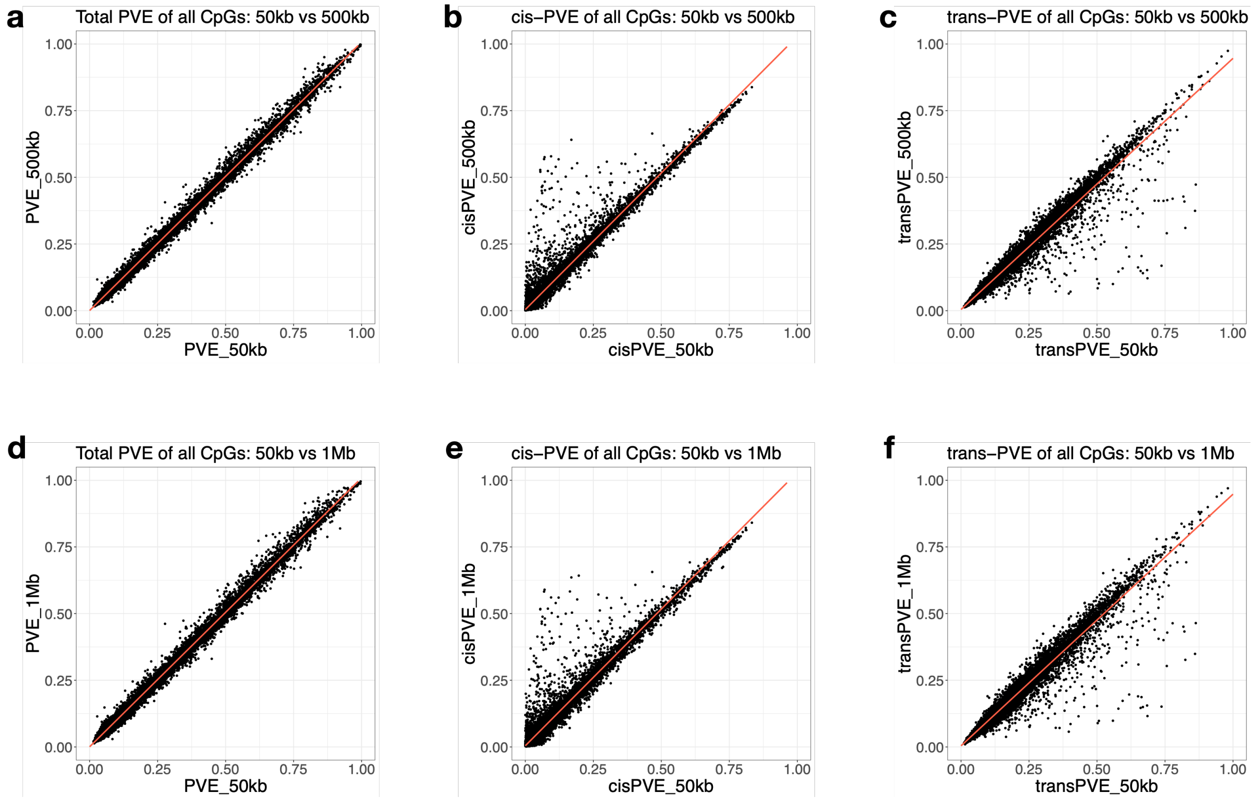

**Supplementary Figure 9. Number of meCpGs with various permutation times at chromosome 22. a** Number of meCpGs identified using different number of permutations on Chr 22. **b** Jaccard index of meCpGs between different permutation times.

**a**

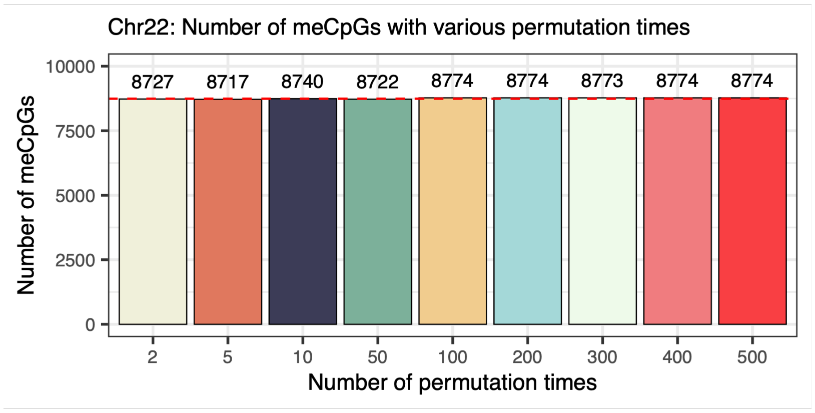


**b**

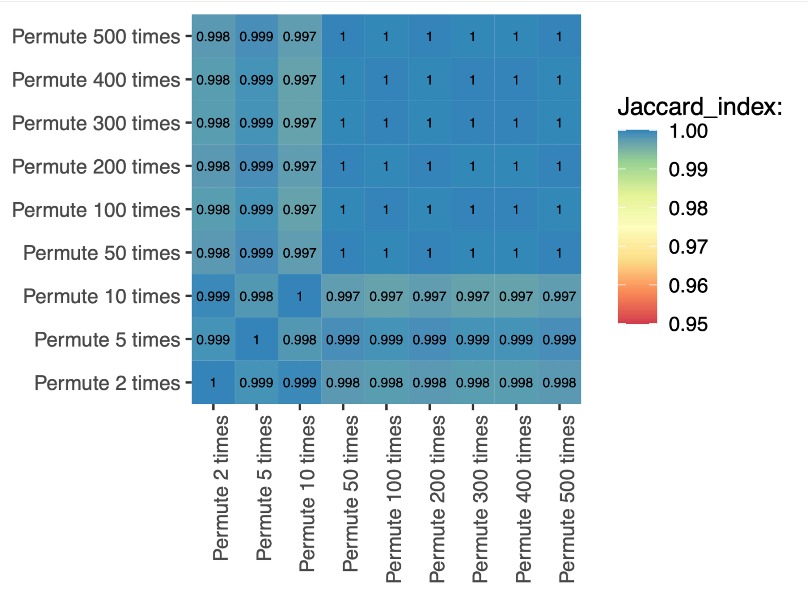

**Supplementary Figure 10. Scatter plot shows the number of CpGs associated with an meQTL.** The x-axis shows the number of CpGs positively associated with an meQTL while the y-axis shows the number of CpGs negatively associated with an meQTL. Color represents the proportion of positively associated CpGs for an meQTL.

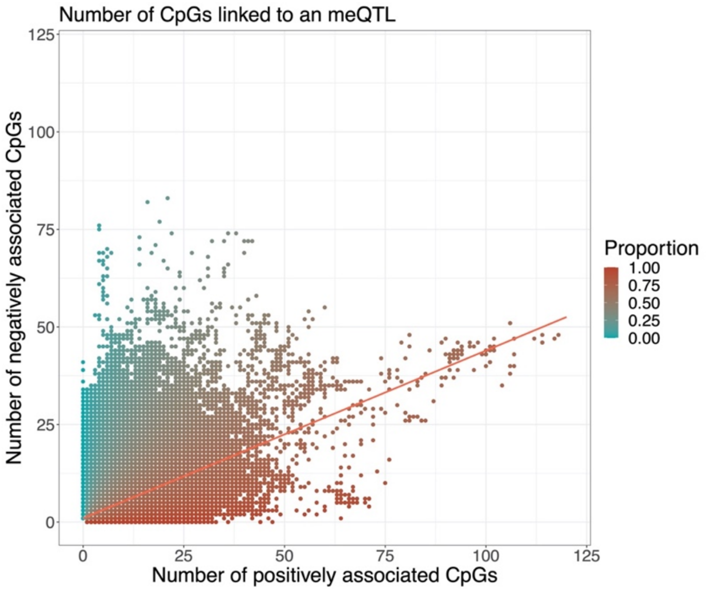

**Supplementary Figure 11. a-b** Comparison of the effect sizes of the meQTLs in the available commonly tested SNP-CpG pairs between GENOA and Hawe et al. x-axis: effect sizes in GENOA; y-axis: effect sizes in Hawe et al. Red color represents the SNP-CpG pairs that are significant in Hawe et al and replicated in GENOA, where the p value threshold in Hawe et al is 0.05/1338027 in European and 0.05/1223921 in South Asian with Bonferroni correction. Blue color represents the SNP-CpG pairs that are significant in Hawe et al but not replicated in GENOA. Association testing in Hawe et al was carried out using Quicktest. The GENOA study test statistics were obtained from two-sided Wald test. **c-d** Boxplot of the SNP allele frequencies of GENOA in comparison with European and South Asian populations in the Hawe et al study for the replicated meQTLs (red) and unreplicated meQTLs (blue). In the boxplots, the center line, box limits and whiskers denote the median, upper and lower quartiles, and 1.5× interquartile range, respectively. **e-f** Density plot of SNP allele frequencies of GENOA in comparison with the European and South Asian populations in the Hawe et al study for the replicated meQTLs (red) and unreplicated meQTLs (blue). Statistics were computed based on a sample size of n = 3,799 in European and 3,195 South Asian participants in Hawe et al, and a sample size of n = 961 in GENOA.

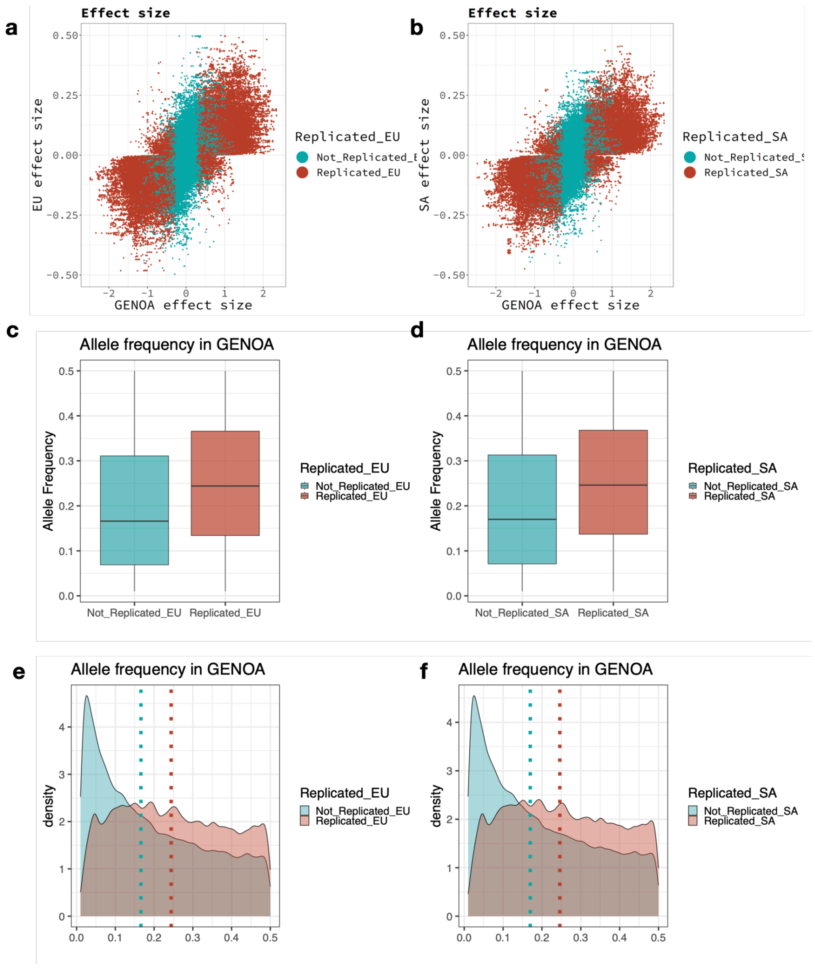

**Supplementary Figure 12. a** Comparison of the effect sizes of the meQTLs in the available commonly tested SNP-CpG pairs between GENOA and BEST studies. x-axis: effect sizes in GENOA; y-axis: effect sizes in BEST. Red color represents the SNP-CpG pairs that are significant in BEST and replicated in GENOA, where the p value threshold in BEST is 0.05/8395616 with Bonferroni correction. Blue color represents the SNP-CpG pairs that are significant in BEST but not replicated in GENOA. The BEST study used Pearson product-moment correlation coefficients and beta distribution-adjusted empirical p-values obtained from FastQTL. The GENOA study test statistics were obtained from two-sided Wald test. **b** Comparison of the allele frequencies of GENOA for the replicated meQTLs (red) and unreplicated meQTLs (blue). In the boxplots, the center line, box limits and whiskers denote the median, upper and lower quartiles, and 1.5× interquartile range, respectively. **c** Density plot of SNP allele frequencies in GENOA for replicated meQTLs (red) and unreplicated meQTLs (blue). Statistics were computed based on a sample size of n = 337 in BEST, and a sample size of n = 961 in GENOA.

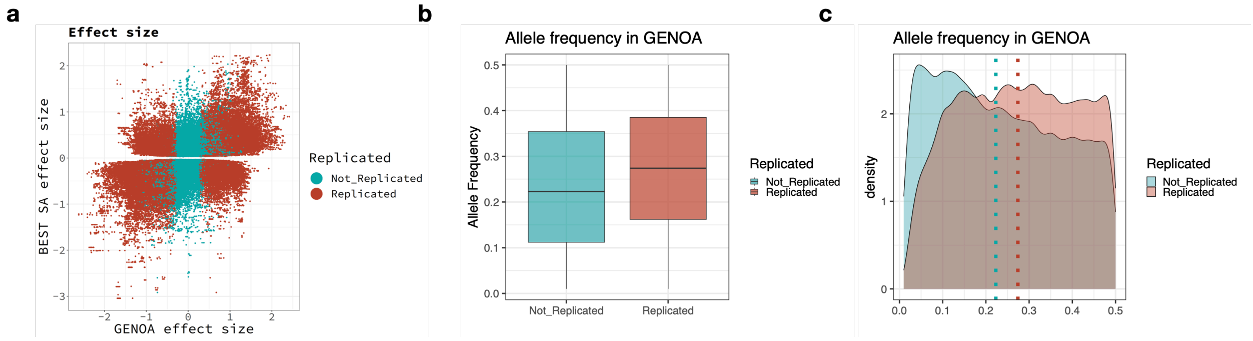

**Supplementary Figure 13. a** Comparison of the effect sizes of the meQTLs in the available commonly tested SNP-CpG pairs between GENOA and GoDMC studies. x-axis: effect sizes in GENOA; y-axis: effect sizes in GoDMC. Red color represents the SNP-CpG pairs that are significant in GoDMC and replicated in GENOA, where the p value threshold in GoDMC is 0.05/9010077 with Bonferroni correction. Blue color represents the SNP-CpG pairs that are significant in GoDMC but not replicated in GENOA. The test statistics in GoDMC study were obtained from two-sided meta-analyses, and the test statistics in GENOA study were obtained from two-sided Wald test. **b** Comparison of the allele frequencies in GENOA for the replicated meQTLs (red) and unreplicated meQTLs (blue). In the boxplots, the center line, box limits and whiskers denote the median, upper and lower quartiles, and 1.5× interquartile range, respectively. **c** Density plot of SNP allele frequencies in GENOA for the replicated meQTLs (red) and unreplicated meQTLs (blue). Statistics were computed based on a sample size of n = 27,750 in GoDMC, and a sample size of n = 961 in GENOA.

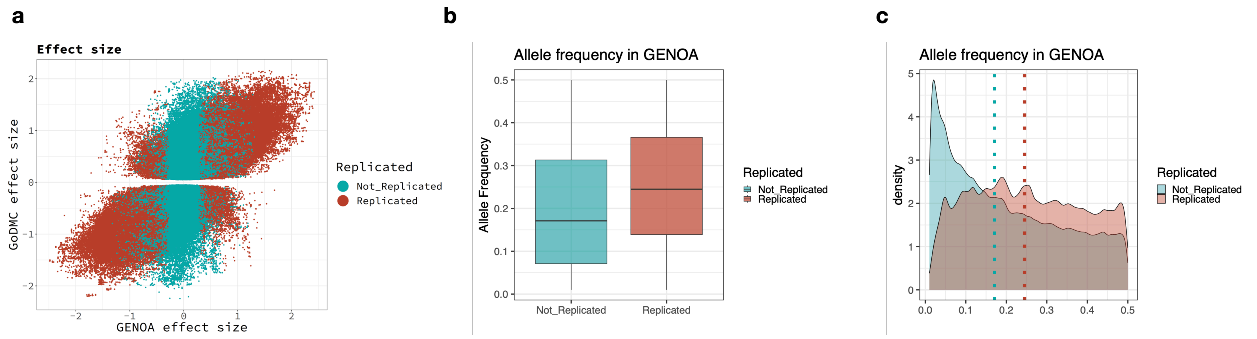

**Supplementary Figure 14. Methylation level heritability estimation and partitioning.** The meCpG sites have higher median PVE (SNP heritability, 24.64%) than the non meCpG sites (6.57%). Statistics were computed based on a sample size of n = 961 and for 728,578 CpG sites.

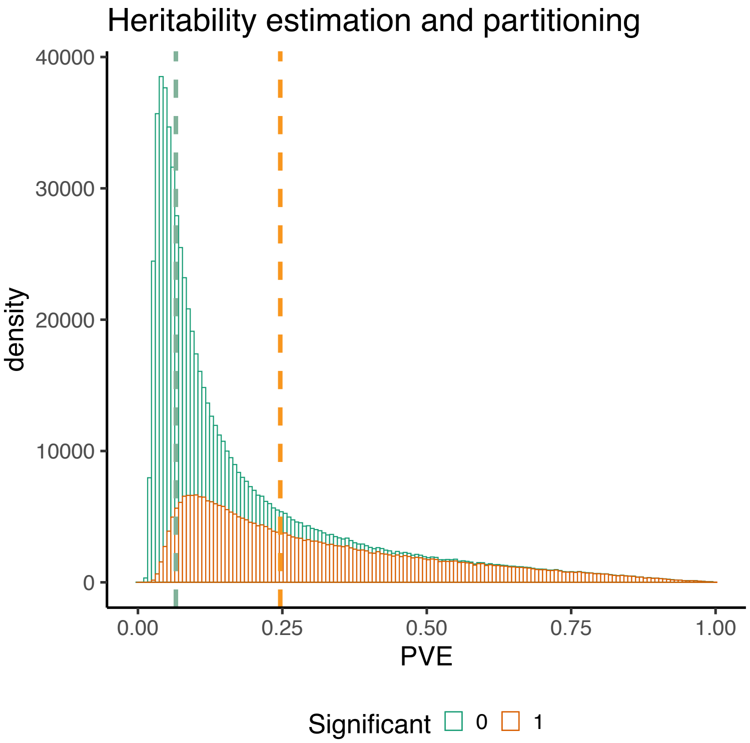

**Supplementary Figure 15. Violin Plot shows the proportion of cis-SNP heritability explained by identified independent meQTLs in meCpGs.** Results are obtained through BSLMM. The identified primary and all independent meQTLs generally explains a large proportion of cis-SNP heritability. In the boxplots, the center line, box limits and whiskers denote the median, upper and lower quartiles, and 1.5× interquartile range, respectively. Statistics were computed based on a sample size of n = 961 and for 320,965 meCpG sites.

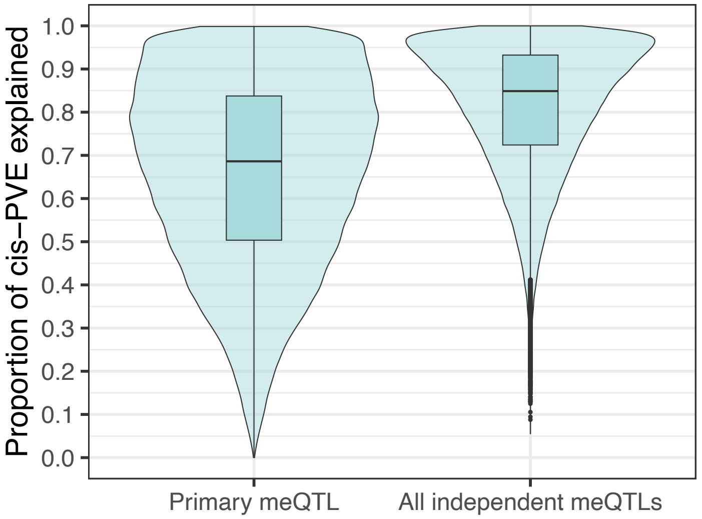

**Supplementary Figure 16. The proportion of cis-SNP heritability for methylation explained by identified primary meQTLs depends on the number of meQTLs identified.** Violin plots show the proportion of cis-SNP heritability explained by identified primary meQTLs (y-axis) with respect to the number of identified independent meQTLs (x-axis) in meCpGs. In the boxplots, the center line, box limits and whiskers denote the median, upper and lower quartiles, and 1.5× interquartile range, respectively. Statistics were computed based on a sample size of n = 961 and for 320,965 meCpG sites.

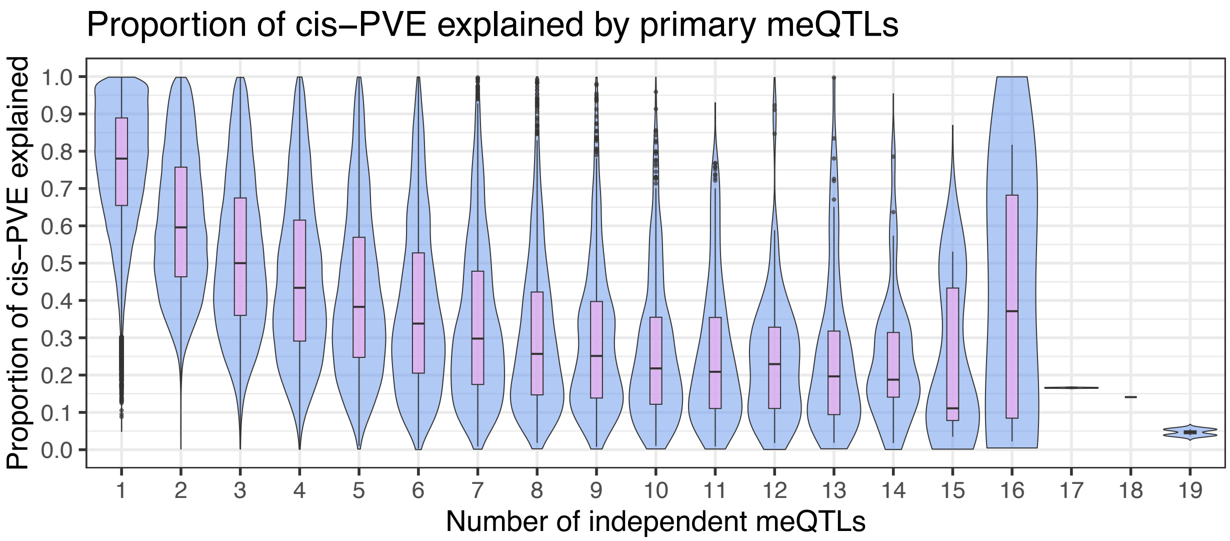

**Supplementary Figure 17. The proportion of cis-SNP heritability for methylation explained by identified independent meQTLs depends on the number of meQTLs identified.** Violin plots show the proportion of cis-SNP heritability explained by all independent meQTLs with respect to the number of identified independent meQTLs (x-axis) in meCpGs. In the boxplots, the center line, box limits and whiskers denote the median, upper and lower quartiles, and 1.5× interquartile range, respectively. Statistics were computed based on a sample size of n = 961 and for 320,965 meCpG sites.

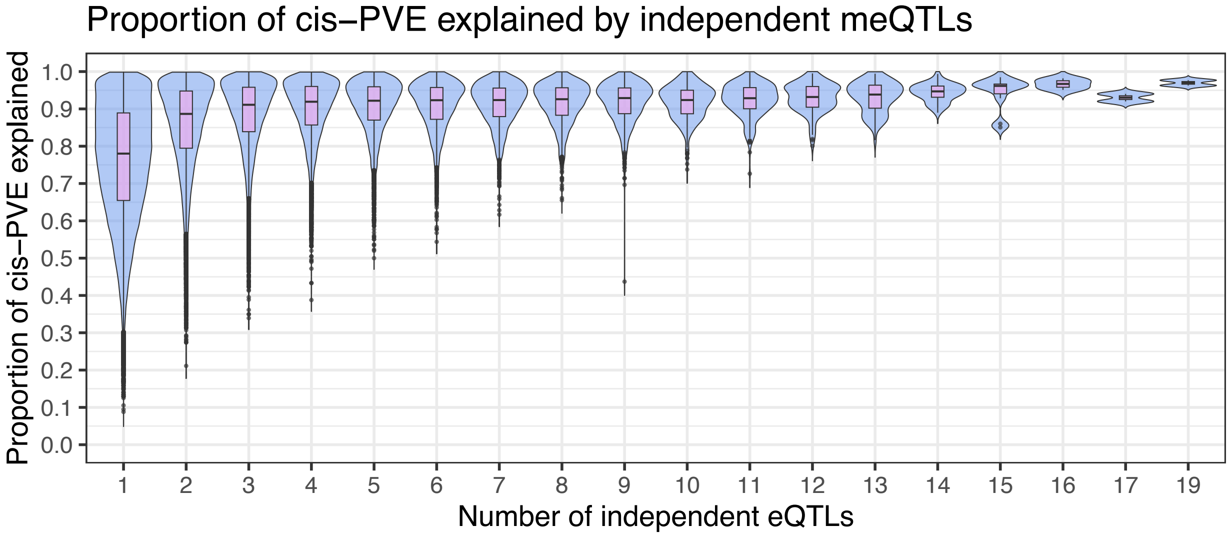

**Supplementary Figure 18. Enrichment results from two functional enrichment tests on meQTLs**. **a** Results from the two-sided Fisher's exact test. **b** Results from Torus. Bar graphs show odds ratios; error bars show 95% confidence intervals. Statistics were computed based on a sample size of n =961 and for 320,965 meCpG sites.

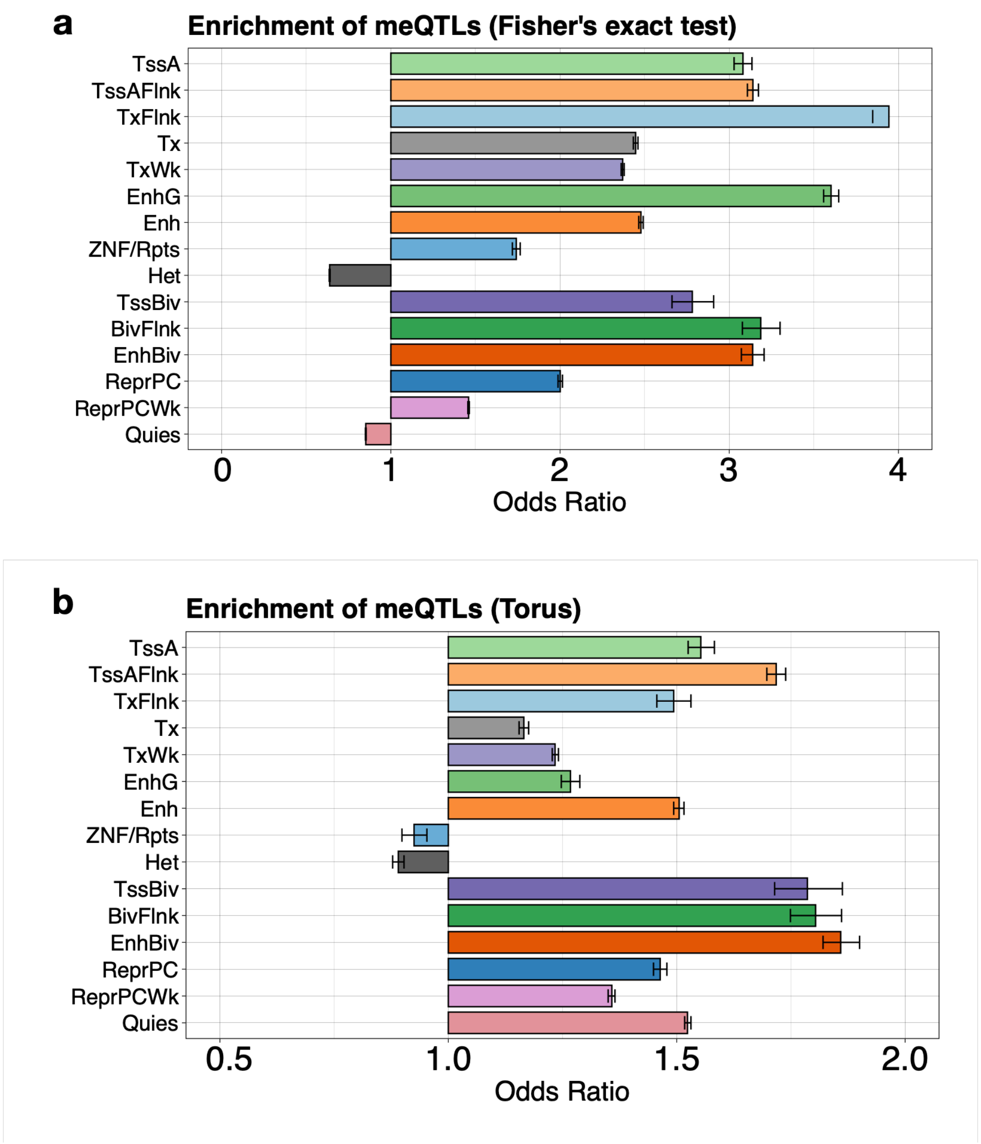

**Supplementary Figure 19. Enrichment odds ratio of meCpGs with one or more independent meQTLs together with the 95% CI. a** Enrichment odds ratio of meCpGs with one independent meQTL together with the 95% CI computed in different annotated genomic regions. **b** Enrichment odds ratio of meCpGs with more than one independent meQTLs together with the 95% CI computed in different annotated genomic regions. The meCpG sites with only one independent meQTL are depleted in CpG island shores and enriched in shelves, but the meCpG sites with more than one independent meQTLs are enriched in CpG island shores and depleted in shelves. Bar graphs show odds ratios; error bars show 95% confidence intervals. Statistics were computed based on a sample size of n =961 and for 320,965 meCpG sites.

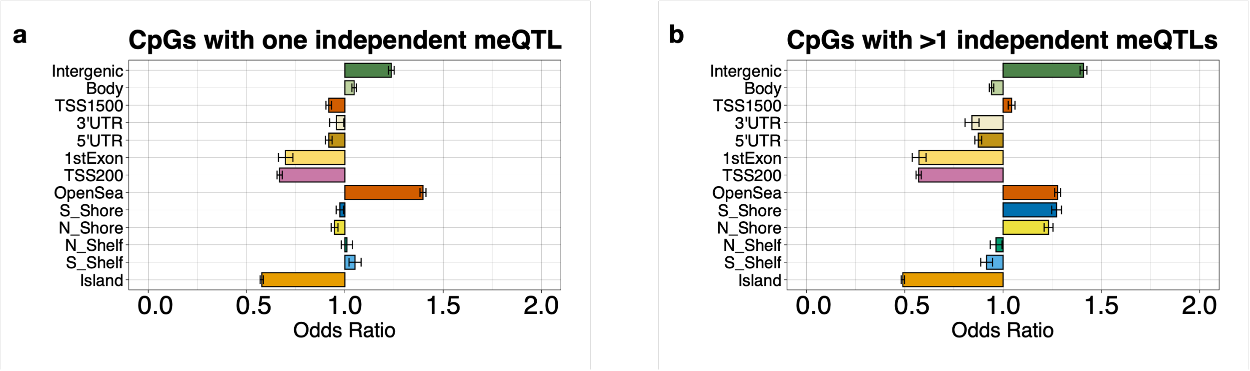

**Supplementary Figure 20. Compare SEM and SME model in mediation analysis.** The -log10 p values in the SEM and SME two-sided Sobel tests for same eGene-meCpG pairs are largely consistent. SME: SNP-Methylation-Expression direction; SEM: SNP-Expression-Methylation direction.

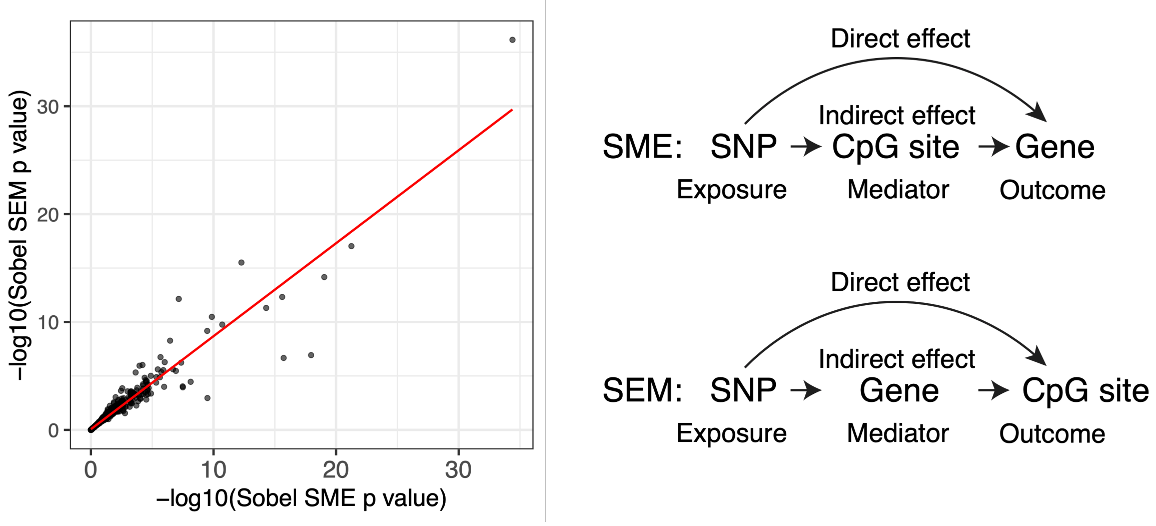

**Supplementary Figure 21. Effect of choice of prior probability ($p_{12}$) of common causal variant (CCV) on the posterior probability of $p_{12}$. a-f** For each eGene-meCpG pair tested in colocalization analyses, we plotted the relative posterior support for a CCV (as opposed to distinct causal variants, DCV) for each colocalization test, defined by posterior probability (PP) of CCV/(PP of DCV + PP of CCV), against the number of SNPs used for each co-localization test . The red line is the Loess smoothing curve of these points. The black line is the relative prior support for a CCV, based on the values selected the priors ($p_1$, $p_2$, and $p_{12}$), calculated following Guo et al [1]. This data is restricted to eGene-meCpG pairs with posterior probability for a CCV to be greater than 0.8. The error band indicates 95% confidence interval.

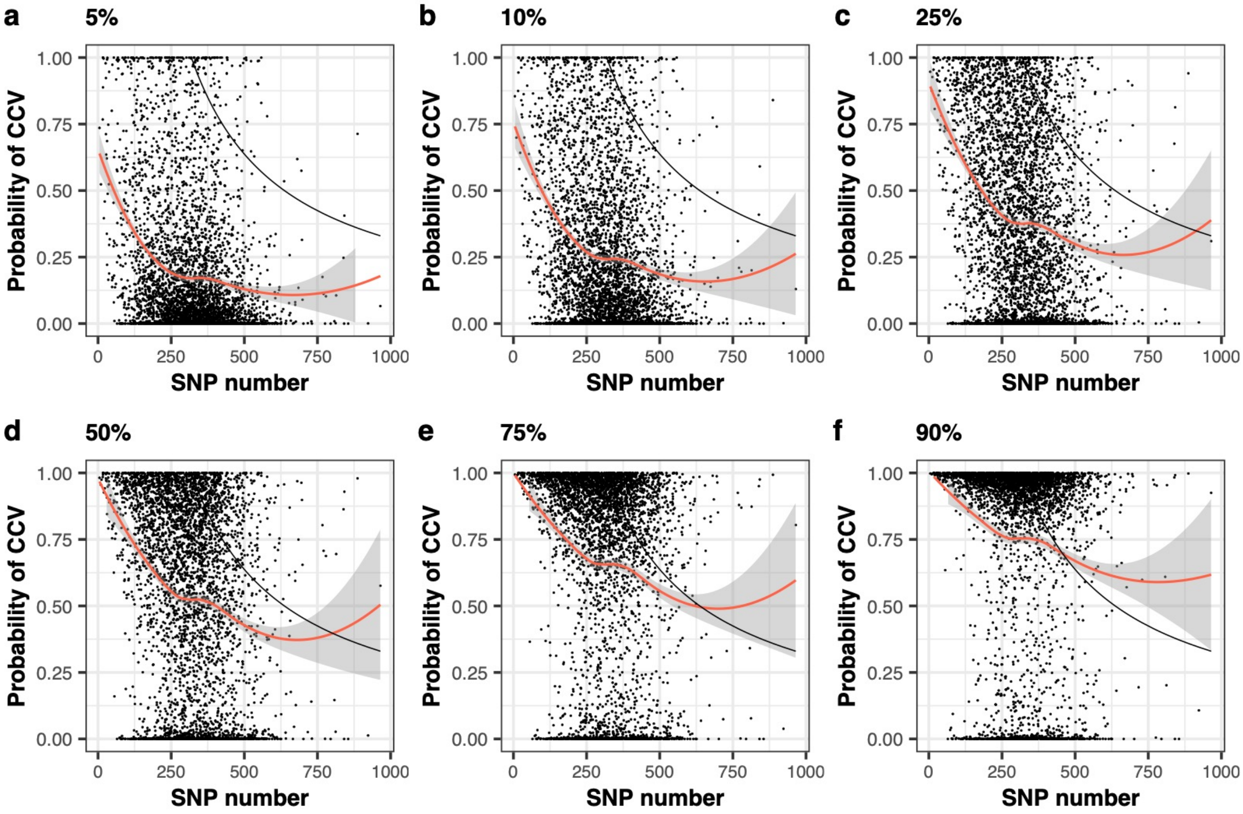

## Supplementary Tables

**Supplementary Table 1. Summary of cis-eQTL and cis-meQTL signals identified in genome-wide analyses.** The first column shows the summary statistics in the meQTL study, the second column shows the summary statistics in the eQTL study.

| Items | cis-meQTL analysis (n=961) | cis-eQTL analysis (n=1032) |
|---|---|---|
| Tests conducted | 221,531,844 | 14,511,338 |
| Significant meCpGs/eGenes | 320,965 | 5,475 |
| Total meQTLs/eQTLs | 4,565,687 | 354,931 |
| Unique primary meQTLs/eQTLs | 254,113 | 5,406 |

**Supplementary Table 2. Heritability estimation with different meQTL mapping window sizes.** We compared the window sizes between 50kb and 1Mb, the heritability estimation are highly consistent with each other.

| Pearson's correlation | Total | Cis | Trans |
|---|---|---|---|
| PVE correlation | 0.998 | 0.978 | 0.979 |
| PVE correlation p value (Two-sided t test) | <2.23e-308 | <2.23e-308 | <2.23e-308 |

**Supplementary Table 3. Comparison of cis-meQTL results with previous studies.** The columns shows the PubMed ID (first column), cis-window size (second column) analyzed in GoDMC (first row), Europeans in Hawe et al. (second row), South Asians in Hawe et al. (third row), FHS (fourth row), BEST (fifth row), PB (sixth row), and CBA (seventh row), the methylation assay used (third column), sample size (fourth column), tissue source (fifth column), ancestry (sixth column), available SNP-CpG pairs in the study that are also tested in GENOA (seventh column), replicate rate (π1) at p value threshold in the original compared study (eighth column), replicate rate (π1) at common p value threshold 1e-5 in both studies (ninth column), and replicate rate (π1) at Bonferroni corrected p value threshold at FDR 0.05 for available SNP-CpG pairs tested in both studies (tenth column). The test statistics in GENOA study were obtained from two-sided Wald test, and the test statistics in other studies were obtained from the original papers.

| **Studies** | **Pubmed ID** | **cis window size** | **Methylation assay** | **Sample size** | **Tissue source** | **Ancestry** | **Available SNP-CpG pairs also tested in GENOA** | **Replicate rate ($\pi_1$) at p value threshold in original study** | **Replicate rate ($\pi_1$) at p value threshold 1e-5** | **Replicate rate ($\pi_1$) at Bonferroni corrected p value threshold at FDR 0.05 for available SNP-CpG pairs tested in both studies** |
|---|---|---|---|---|---|---|---|---|---|---|
| **Genetics of DNA Methylation Consortium. (GoDMC)** [2] | 34493871 | <1Mb | Illumina HumanMethylation450 BeadChip | 27,750 | Whole blood | European | 9,010,077 | 0.77 (p<1e-8) | 0.76 (p<1e-5) | 0.77 (p<0.05/9010077) |
| **Hawe et al. 2022** [3] | 34980917 | <1Mb | Illumina HumanMethylation450 BeadChip | 3,799 | Peripheral blood | European | 2,304,374 | 0.93 (p<1e-14) | 0.91 (p<1e-5) | 0.92 (p<0.05/2304374) |
| **Hawe et al. 2022** [3] | 34980917 | <1Mb | Illumina HumanMethylation450 BeadChip | 3,195 | Peripheral blood | South Asian | 2,304,374 | 0.9 (p<1e-14) | 0.9 (p<1e-5) | 0.9 (p<0.05/2304374) |
| **Framingham Heart Study (FHS)** [4] | 31537805 | <1Mb | Illumina HumanMethylation450 BeadChip | 4,170 | Whole blood buffy coat | European | 4,951,955 | 0.82 (p<2e-11) | 0.82 (p<1e-5) | 0.82 (p<0.05/4951955) |
| **Bangladesh Vitamin E and Selenium Trial (BEST) study** [5] | 29476079 | <500kb | Illumina HumanMethylation450 BeadChip | 337 | Whole blood | South Asian (Bangladesh) | 8,395,616 | 0.91 | 0.9 (p<1e-5) | 0.93 (p<0.05/8395616) |
| **Adult peripheral blood (PB)** [6] | 24555763 | <50kb | Illumina HumanMethylation27 BeadChip | 90 | Peripheral blood | African American | 517 | 0.98 | 0.98 | 0.98 (p<0.05/517) |
| **Umbilical cord blood at birth (CBA) samples** [6] | 24555763 | <50kb | Illumina HumanMethylation27 BeadChip | 87 | Cord blood | African American | 531 | 0.98 | 0.98 | 0.98 (p<0.05/531) |

**Supplementary Table 4. The number of independent meQTLs identified in meCpGs through the conditional analysis.** This table lists the number of meCpGs that contain different numbers of independent meQTLs (columns).

| | | | | | | | |
|---|---|---|---|---|---|---|---|
| Number of independent meQTLs | **1** | **2** | **3** | **4** | **5** | **6** | **7** |
| Number of meCpG sites | 176,420 | 74508 | 34114 | 16700 | 8649 | 4730 | 2605 |
| Number of independent meQTLs | **8** | **9** | **10** | **11** | **12** | **13** | **14** |
| Number of meCpG sites | 1444 | 791 | 466 | 263 | 139 | 78 | 38 |
| Number of independent meQTLs | **15** | **16** | **17** | **18** | **19** | | |
| Number of meCpG sites | 11 | 4 | 2 | 1 | 2 | | |

**Supplementary Table 5. Number of eGene-meCpG pairs with probability of a common causal variant for various values of the prior $p_{12}$.** Colocalized pairs are restricted to eGene-meCpG pairs with posterior probability for colocalization to be greater than 0.8.

| | Prior probability that an eQTL is an meQTL | Prior ($p_{12}$) | Number of eGene-meCpG pairs with PP4>80% | Proportion of eGene-meCpG pairs with PP4>80% |
|---|---|---|---|---|
| Colocalization | 90% | 7.56e-04 | 2859 | 58.9% |
| | 75% | 6.3e-04 | 2,264 | 46.27% |
| | 50% | 4.2e-04 | 1429 | 29.44% |
| | 25% | 2.1e-04 | 750 | 15.45% |
| | 10% | 8.4e-05 | 373 | 7.68% |
| | 5% | 4.2e-05 | 244 | 5.03% |
| Total number of eGene-meCpG pairs tested for colocalization was 4,854. | | | | |

**Supplementary Table 6. Colocalization analysis between eQTL, meQTL and GWAS traits.** The GWAS traits include SBP, PP, DBP, HTN, T2D, and BMI. This table lists the colocalization between meQTL and GWAS traits with default settings in coloc (first row), meQTL and GWAS traits with susie (second row), eQTL and GWAS traits with default settings in coloc (third row), eQTL and GWAS traits with susie (fourth row), and meQTL-eQTL-GWAS with moloc (fifth row).

| Colocalization method | SBP | BMI | DBP | HTN | PP | T2D |
|---|---|---|---|---|---|---|
| **gwas_meQTL_coloc_default** | 0 | 1 | 2 | 0 | 0 | 1 |
| **gwas_meQTL_coloc_susie** | 0 | 1 | 1 | 1 | 3 | 0 |
| **gwas_eQTL_coloc_default** | 0 | 2 | 1 | 1 | 1 | 1 |
| **gwas_eQTL_coloc_susie** | 0 | 2 | 1 | 2 | 4 | 1 |
| **gwas_eqtl_meqtl_moloc** | 1 | 0 | 2 | 0 | 1 | 0 |

**Supplementary Table 7. Colocalized pairs between eQTL, meQTL and GWAS traits.** The GWAS traits include SBP, PP, DBP, HTN, T2D, and BMI. This table lists the colocalized pairs between meQTL, eQTL and GWAS traits (first column), with different colocalization method (second column), and the corresponding eGenes (third column), meCpG (fourth column), SNP (fifth column), and the posterior probability PPA (sixth column) of the SNP is associated with eQTL, meQTL and GWAS trait with the moloc method, the posterior probability PP4 of the SNP is associated with eQTL and GWAS with coloc (seventh column), the posterior probability PP4 of the SNP is associated with meQTL and GWAS with coloc (eighth column), the posterior probability PP4 of the SNP is associated with eQTL and GWAS with Susie (ninth column), the posterior probability PP4 of the SNP is associated with meQTL and GWAS with Susie (tenth column).

| Trait | Colocalization method | Gene | CpG | SNP | Moloc PPA | Coloc Eqtl Gwas PP4 | Coloc Mqtl gwas PP4 | Susie Eqtl gwas PP4 | Susie MeQTL gwas PP4 |
|---|---|---|---|---|---|---|---|---|---|
| SBP | gwas_eqtl_meqtl_moloc | PPIA | cg13400493 | rs4724322 | 0.955 | 0.697 | 9.99E-01 | 0.071 | 0.955 |
| BMI | gwas_meQTL_coloc | ADCY3 | cg22495460 | rs6717671 | 0.948 | 0.665 | 8.81E-01 | 0.816 | 0.948 |
| BMI | gwas_meQTL_susie | DLG2 | cg21409491 | rs7925739 | 0.895 | 0.488 | 9.48E-01 | 0.139 | 0.895 |
| BMI | gwas_eQTL_coloc | ADCY3 | cg22495460 | rs6717671 | 0.948 | 0.665 | 8.81E-01 | 0.816 | 0.948 |
| BMI | gwas_eQTL_coloc | DLG2 | cg21409491 | rs7925739 | 0.895 | 0.488 | 9.48E-01 | 0.139 | 0.895 |
| BMI | gwas_eQTL_susie | DLG2 | cg21409491 | rs7925739 | 0.895 | 0.488 | 9.48E-01 | 0.139 | 0.895 |
| BMI | gwas_eQTL_susie | ZNF434 | cg01273991 | rs117554633 | 0.87 | 0.766 | 6.73E-03 | 0.02 | 0.87 |
| DBP | gwas_meQTL_coloc | ULK4 | cg05589743 | rs2272007 | 0.976 | 0.813 | 1.05E-07 | 0.953 | 0.976 |
| DBP | gwas_meQTL_coloc | PBX3 | cg16144450 | rs631287 | 0.008 | 0.0009 | 4.48E-03 | 0.975 | 0.008 |
| DBP | gwas_meQTL_susie | PBX3 | cg16144450 | rs631287 | 0.008 | 0.0009 | 4.48E-03 | 0.975 | 0.008 |
| DBP | gwas_eQTL_coloc | ULK4 | cg05589743 | rs2272007 | 0.976 | 0.813 | 1.05E-07 | 0.953 | 0.976 |
| DBP | gwas_eQTL_susie | ULK4 | cg05589743 | rs2272007 | 0.976 | 0.813 | 1.05E-07 | 0.953 | 0.976 |
| DBP | gwas_eqtl_meqtl_moloc | ULK4 | cg05589743 | rs2272007 | 0.976 | 0.813 | 1.05E-07 | 0.953 | 0.976 |
| DBP | gwas_eqtl_meqtl_moloc | PPIA | cg13400493 | rs4724322 | 0.955 | 0.697 | 9.99E-01 | 0.073 | 0.955 |
| HTN | gwas_meQTL_susie | RBM47 | cg18266414 | rs113869309 | 0.901 | 0.643 | 1E+00 | 0.028 | 0.901 |
| HTN | gwas_eQTL_coloc | GINS2 | cg14451446 | rs12149168 | 0.9997 | 0.999 | 7.71E-01 | 0.319 | 0.9997 |
| HTN | gwas_eQTL_susie | RBM47 | cg18266414 | rs113869309 | 0.901 | 0.643 | 1E+00 | 0.028 | 0.901 |
| HTN | gwas_eQTL_susie | GINS2 | cg14451446 | rs12149168 | 0.9997 | 0.999 | 7.71E-01 | 0.319 | 0.9997 |
| PP | gwas_meQTL_susie | AP3D1 | cg01292427 | rs10413398 | 0.984 | 0.902 | 1E+00 | 0.793 | 0.984 |
| PP | gwas_meQTL_susie | DOT1L | cg27654029 | rs73916864 | 0.921 | 0.876 | 1E+00 | 0.436 | 0.921 |
| PP | gwas_meQTL_susie | PLEKHJ1 | cg10746224 | rs2238595 | 0.98 | 0.844 | 1E+00 | 0.106 | 0.98 |
| PP | gwas_eQTL_coloc | AP3D1 | cg01292427 | rs10413398 | 0.984 | 0.902 | 1E+00 | 0.793 | 0.984 |
| PP | gwas_eQTL_susie | TRIP4 | cg13416889 | rs673931 | 0.99999 | 0.9999 | 9.33E-01 | 0.377 | 0.99999 |
| PP | gwas_eQTL_susie | AP3D1 | cg01292427 | rs10413398 | 0.984 | 0.902 | 1E+00 | 0.793 | 0.984 |

| | | | | | | | | | |
|---|---|---|---|---|---|---|---|---|---|
| PP | gwas_eQTL_susie | DOT1L | cg27654029 | rs73916864 | 0.921 | 0.876 | 1E+00 | 0.436 | 0.921 |
| PP | gwas_eQTL_susie | PLEKHJ1 | cg10746224 | rs2238595 | 0.98 | 0.844 | 1E+00 | 0.106 | 0.98 |
| PP | gwas_eqtl_meqtl_moloc | AP3D1 | cg01292427 | rs10413398 | 0.984 | 0.902 | 1E+00 | 0.793 | 0.984 |
| T2D | gwas_meQTL_coloc | PTGES2 | cg04745703 | rs9695803 | 0.999 | 0.988 | 4.94E-01 | 0.956 | 0.999 |
| T2D | gwas_eQTL_coloc | PTGES2 | cg04745703 | rs9695803 | 0.999 | 0.988 | 4.94E-01 | 0.956 | 0.999 |
| T2D | gwas_eQTL_susie | C6orf170 | cg21760990 | rs9490173 | 0.977 | 0.821 | 1E+00 | 0.014 | 0.977 |

**Supplementary Table 8. Comparison of colocalization and multi-traits colocalization results for GWAS with African ancestry and GWAS with European ancestry.**

| Ancestry | African ancestry GWAS | | | European ancestry GWAS | | |
|---|---|---|---|---|---|---|
| Traits | SBP | BMI | DBP | SBP | BMI | DBP |
| GWAS+meQTL | 0 | 1 | 2 | 0 | 2 | 3 |
| GWAS+eQTL | 0 | 2 | 1 | 0 | 2 | 0 |
| GWAS+meQTL+eQTL | 1 | 0 | 2 | 0 | 1 | 0 |

**Supplementary Table 9. Summary of trans-meQTL results for 500 randomly selected CpG sites on chr 22.** The test statistics in GENOA study were obtained from two-sided Wald test.

| Window size | Total tested pairs for 500 CpGs | Same chromosome | | | Other chromosomes | | |
|---|---|---|---|---|---|---|---|
| | | Version 1: p value < 5*10-8 | Version 2: Bonferroni for pairs within each CpG | Version 3: Bonferroni for all pairs whole genome | Version 1: p value < 5*10-8 | Version 2: Bonferroni for pairs within each CpG | Version 3: Bonferroni for all pairs whole genome |
| **>50kb** | 4834161450 | 6948 | 5631 | 2334 | 0 | 0 | 0 |
| **>1Mb** | 4296725475 | 743 | 419 | 137 | 0 | 0 | 0 |

## Supplementary References:


1 Guo, H. *et al.* Integration of disease association and eQTL data using a Bayesian colocalisation approach highlights six candidate causal genes in immune-mediated diseases. *Hum Mol Genet* **24**, 3305-3313, doi:10.1093/hmg/ddv077 (2015).

2 Min, J. L. *et al.* Genomic and phenotypic insights from an atlas of genetic effects on DNA methylation. *Nat Genet* **53**, 1311-1321, doi:10.1038/s41588-021-00923-x (2021).

3 Hawe, J. S. *et al.* Genetic variation influencing DNA methylation provides insights into molecular mechanisms regulating genomic function. *Nature Genetics* **54**, 18-+, doi:10.1038/s41588-021-00969-x (2022).

4 Huan, T. *et al.* Genome-wide identification of DNA methylation QTLs in whole blood highlights pathways for cardiovascular disease. *Nat Commun* **10**, 4267, doi:10.1038/s41467-019-12228-z (2019).

5 Pierce, B. L. *et al.* Co-occurring expression and methylation QTLs allow detection of common causal variants and shared biological mechanisms. *Nat Commun* **9**, 804, doi:10.1038/s41467-018-03209-9 (2018).

6 Bradley, R. G. *et al.* Influence of child abuse on adult depression: moderation by the corticotropin-releasing hormone receptor gene. *Arch Gen Psychiatry* **65**, 190-200, doi:10.1001/archgenpsychiatry.2007.26 (2008).
