# Supplementary material for: meQTL mapping in the GENOA study reveals genetic determinants of DNA methylation in African Americans
Source: Nat Commun. 2023 May 11;14:2711. doi: 10.1038/s41467-023-37961-4 (PMC10175543; doi:10.1038/s41467-023-37961-4)
Supplement: Supplementary file 3 — Reporting Summary [file 41467_2023_37961_MOESM3_ESM.pdf]

nature portfolio

Corresponding author(s): Xiang Zhou, Jennifer Smith

Last updated by author(s): Mar 4, 2023

Reporting Summary

Nature Portfolio wishes to improve the reproducibility of the work that we publish. This form provides structure for consistency and transparency in reporting. For further information on Nature Portfolio policies, see our Editorial Policies and the Editorial Policy Checklist.

Statistics

For all statistical analyses, confirm that the following items are present in the figure legend, table legend, main text, or Methods section.

n/a	Confirmed	
☐	☒	The exact sample size (*n*) for each experimental group/condition, given as a discrete number and unit of measurement
☐	☒	A statement on whether measurements were taken from distinct samples or whether the same sample was measured repeatedly
☐	☒	The statistical test(s) used AND whether they are one- or two-sided<br>*Only common tests should be described solely by name; describe more complex techniques in the Methods section.*
☐	☒	A description of all covariates tested
☐	☒	A description of any assumptions or corrections, such as tests of normality and adjustment for multiple comparisons
☐	☒	A full description of the statistical parameters including central tendency (e.g. means) or other basic estimates (e.g. regression coefficient) AND variation (e.g. standard deviation) or associated estimates of uncertainty (e.g. confidence intervals)
☐	☒	For null hypothesis testing, the test statistic (e.g. *F*, *t*, *r*) with confidence intervals, effect sizes, degrees of freedom and *P* value noted<br>*Give P values as exact values whenever suitable.*
☐	☒	For Bayesian analysis, information on the choice of priors and Markov chain Monte Carlo settings
☒	☐	For hierarchical and complex designs, identification of the appropriate level for tests and full reporting of outcomes
☐	☒	Estimates of effect sizes (e.g. Cohen's *d*, Pearson's *r*), indicating how they were calculated

Our web collection on statistics for biologists contains articles on many of the points above.

Software and code

Policy information about availability of computer code

Data collection	No software was used in data collection.
Data analysis	We used the following open source softwares:<br>R version 3.6.0: data processing, computing and plotting figures<br>SHAPEIT version v2.r: genotype imputation<br>IMPUTE version 2: genotype imputation<br>GEMMA version 0.98.1: estimate relatedness matrix, perform association testing, calculate heritability<br>PLINK version v1.07: extracting subsets of genotype data<br>GENESIS R package version 2.28.0: infer population structure<br>shinyMethyl R package version 1.34.0: generate the density plot of the raw intensity data<br>ENmix R package version 1.34.0: sample QC<br>Minfi R package version 1.44.0: background correction and normalization<br>IlluminaHumanMethylationEPICanno.ilm10b2.hg19 R package version 0.6.0: annotation for Illumina's EPIC methylation arrays<br>Torus package version 1.0: enrichment analysis<br>coloc R package version 5.1.0: colocalization analysis<br>moloc R package version 0.1.0: multi-trait colocalization analysis<br>lme4 R package version 1.1.31: fit linear mixed model<br>sva R package version 3.38.0: batch effect removal in gene expression

For manuscripts utilizing custom algorithms or software that are central to the research but not yet described in published literature, software must be made available to editors and reviewers. We strongly encourage code deposition in a community repository (e.g. GitHub). See the Nature Portfolio guidelines for submitting code & software for further information.

Data

Policy information about availability of data

All manuscripts must include a data availability statement. This statement should provide the following information, where applicable:

- Accession codes, unique identifiers, or web links for publicly available datasets
- A description of any restrictions on data availability
- For clinical datasets or third party data, please ensure that the statement adheres to our policy

The methylation data generated in this study have been deposited in the Gene Expression Omnibus (GEO) database under accession code GSE210256 [https://www.ncbi.nlm.nih.gov/geo/query/acc.cgi?acc=GSE210256].
The SNP data are available in the Database of Genotypes and Phenotypes (dbGaP) under accession number phs001238.v2.p1 [https://www.ncbi.nlm.nih.gov/projects/gap/cgi-bin/study.cgi?study_id=phs001238.v2.p1] under restricted access due to IRB, access can be obtained by written request to J.A.S. and S.L.R.K.
The UK Biobank data are from UK Biobank resource under application number 30686.
The human regulatory motif sites are downloaded from http://motifmap.ics.uci.edu.
The summary statistics from the GoDMC study are available at http://mqtldb.godmc.org.uk.
The summary statistics from Hawe et al are available at https://zenodo.org/record/5196216#.YRZ3TfJxeUk.
The summary statistics from the FHS study are available at https://ftp.ncbi.nlm.nih.gov/eqtl/original_submissions/FHS_meQTLs.
The summary statistics from the PB and CBA samples are available at https://static-content.springer.com/esm/art%3A10.1186%2F1471-2164-15-145/MediaObjects/12864_2013_5906_MOESM1_ESM.csv.
The summary statistics from the BEST study are available at https://datadryad.org/stash/dataset/doi:10.5061/dryad.hq68q.
The summary statistics from the GENOA eQTL mapping analysis are available at http://www.xzlab.org/data.html.
The gene expression data used in the GENOA eQTL mapping analysis is available at the Gene Expression Omnibus (GEO) database under accession codes GSE138914 [https://www.ncbi.nlm.nih.gov/geo/query/acc.cgi?acc=GSE138914] for AA and GSE49531 [https://www.ncbi.nlm.nih.gov/geo/query/acc.cgi?acc=GSE49531] for EA.
The summary statistics (mapped to Genome Assembly GRCh37) generated in the GENOA meQTL mapping analysis are available at http://www.xzlab.org/data.html and https://doi.org/10.5281/zenodo.769750993. Source data are provided with this paper.

Human research participants

Policy information about studies involving human research participants and Sex and Gender in Research.

Reporting on sex and gender	This meQTL mapping study used data from 685 females and 276 males, with ages ranging from 20.5 to 86.7 years (median 57.7).
Population characteristics	This meQTL mapping study used data from a total of 961 African American participants, including 685 females and 276 males, with ages ranging from 20.5 to 86.7 years (median 57.7).
Recruitment	Genetic Epidemiology Network of Arteriopathy (GENOA) is a community-based study of hypertensive sibships that was designed to investigate the genetics of hypertension and target organ damage. GENOA includes non-Hispanic African Americans from Jackson, Mississippi, and non-Hispanic whites from Rochester, Minnesota. This meQTL study only includes African Americans (AA) from Jackson, Mississippi. In Jackson, sibships were recruited through hypertensive probands who had participated in the Atherosclerosis Risk in Communities (ARIC) study (PMID: 2646917). The ARIC cohort in Jackson was a probability sample of 45- to 64-year-old African American residents of that community. In the initial phase of GENOA (Phase I: 1996-2001), all members of sibships containing at least 2 individuals with essential hypertension clinically diagnosed before age 60 were invited to participate, including both hypertensive and normotensive siblings. The exclusion criteria for GENOA included secondary hypertension, alcoholism or drug abuse, pregnancy, insulin-dependent diabetes mellitus, or active malignancy. Eighty percent of AA (N=1,482) from the initial study population returned for the second examination (Phase II: 2001-2005). Demographic information, medical history, clinical characteristics, lifestyle factors, and blood samples were collected in each phase. Written informed consent was obtained from all subjects and approval was granted by participating institutional review boards (University of Michigan, University of Mississippi Medical Center, and Mayo Clinic).
Ethics oversight	Institutional Review Boards at the University of Michigan, University of Mississippi Medical Center, and Mayo Clinic approved this study.

Note that full information on the approval of the study protocol must also be provided in the manuscript.

Field-specific reporting

Please select the one below that is the best fit for your research. If you are not sure, read the appropriate sections before making your selection.

☒ Life sciences ☐ Behavioural & social sciences ☐ Ecological, evolutionary & environmental sciences

For a reference copy of the document with all sections, see nature.com/documents/nr-reporting-summary-flat.pdf

Life sciences study design

All studies must disclose on these points even when the disclosure is negative.

Sample size	The Genetic Epidemiology Network of Arteriopathy (GENOA) study is a community-based study of hypertensive sibships that was designed to investigate the genetics of hypertension and target organ damage. The study includes African Americans (AA) from Jackson, MS. After removing outliers, there were 1,599 AA samples with available genotype data. Genomic DNA was extracted from stored peripheral blood leukocytes that was collected at Phase 1 (N=1106) or Phase 2 (N=304). The meQTL mapping analysis was performed using individuals in GENOA study with both genotype and methylation data (N=961). Pre-study power calculations were not performed, but we aimed to maximize sample size based on the cohorts available and included over 3x more participants than the largest previous meQTL study in African ancestry samples.
Data exclusions	The exclusion criteria for GENOA included secondary hypertension, alcoholism or drug abuse, pregnancy, insulin-dependent diabetes mellitus, or active malignancy. We removed samples, SNPs, and probes according to predetermined criteria. For genotype data, samples were excluded if they had an overall SNP call rate<95% or sex mismatch between genotype and self-report. SNPs were excluded if they had a call rate<95%. For methylation data, sex mismatches or sample outliers were removed, and samples and probes with detection rate<10% were removed.
Replication	We compared our cis-meQTL mapping results to six previous cis-meQTL mapping studies that include the Genetics of DNA Methylation Consortium (GoDMC), Hawe et al., Framingham Heart Study (FHS), a study on adult peripheral blood (PB) samples, and umbilical cord blood at birth (CBA) samples, and the Bangladesh Vitamin E and Selenium Trial (BEST) study. We used the pi1 statistic to assess replication consistency. The replication rates across these studies varied between 0.77 and 0.98, with differences in replication rates due to genetic ancestry, methylation measurement platform, cis window size, types of tissue used, different sample sizes, as well as the applied FDR methods. The approaches and results are described in full, in the main text of the manuscript and the Supplementary Tables that address the respective analyses.
Randomization	This is an observational study. Randomization was not applicable.
Blinding	This is an observational study. Blinding was not applicable.

Reporting for specific materials, systems and methods

We require information from authors about some types of materials, experimental systems and methods used in many studies. Here, indicate whether each material, system or method listed is relevant to your study. If you are not sure if a list item applies to your research, read the appropriate section before selecting a response.

Materials & experimental systems

n/a	Involved in the study	
☒	☐	Antibodies
☒	☐	Eukaryotic cell lines
☒	☐	Palaeontology and archaeology
☒	☐	Animals and other organisms
☒	☐	Clinical data
☒	☐	Dual use research of concern

Methods

n/a	Involved in the study	
☒	☐	ChIP-seq
☒	☐	Flow cytometry
☒	☐	MRI-based neuroimaging
